# C-Terminal Region of MAP7 Domain Containing Protein 3 (MAP7D3) Promotes Microtubule Polymerization by Binding at the C-Terminal Tail of Tubulin

**DOI:** 10.1371/journal.pone.0099539

**Published:** 2014-06-13

**Authors:** Saroj Yadav, Paul J. Verma, Dulal Panda

**Affiliations:** 1 IITB-Monash Research Academy, Indian Institute of Technology Bombay, Mumbai, Maharashtra, India; 2 Centre for Reproduction and Development, Monash Institute of Medical Research, Monash University, Clayton, Victoria, Australia; 3 Department of Biosciences and Bioengineering, Indian Institute of Technology Bombay, Mumbai, Maharashtra, India; Indian Institute of science, India

## Abstract

MAP7 domain containing protein 3 (MAP7D3), a newly identified microtubule associated protein, has been shown to promote microtubule assembly and stability. Its microtubule binding region has been reported to consist of two coiled coil motifs located at the N-terminus. It possesses a MAP7 domain near the C-terminus and belongs to the microtubule associated protein 7 (MAP7) family. The MAP7 domain of MAP7 protein has been shown to bind to kinesin-1; however, the role of MAP7 domain in MAP7D3 remains unknown. Based on the bioinformatics analysis of MAP7D3, we hypothesized that the MAP7 domain of MAP7D3 may have microtubule binding activity. Indeed, we found that MAP7 domain of MAP7D3 bound to microtubules as well as enhanced the assembly of microtubules *in vitro*. Interestingly, a longer fragment MDCT that contained the MAP7 domain (MD) with the C-terminal tail (CT) of the protein promoted microtubule polymerization to a greater extent than MD and CT individually. MDCT stabilized microtubules against dilution induced disassembly. MDCT bound to reconstituted microtubules with an apparent dissociation constant of 3.0±0.5 µM. An immunostaining experiment showed that MDCT localized along the length of the preassembled microtubules. Competition experiments with tau indicated that MDCT shares its binding site on microtubules with tau. Further, we present evidence indicating that MDCT binds to the C-terminal tail of tubulin. In addition, MDCT could bind to tubulin in HeLa cell extract. Here, we report a microtubule binding region in the C-terminal region of MAP7D3 that may have a role in regulating microtubule assembly dynamics.

## Introduction

The involvement of microtubules in various cellular events including intracellular transport of vesicles, cell differentiation, cell motility and mitosis is accredited to their dynamic behavior. Microtubule dynamics is regulated by the combined actions of various proteins, which either promote or decrease the assembly and stability of microtubules [1]–[3]. Microtubule associated proteins (MAPs) that bind along the length of microtubules, known as structural MAPs, constitute one of the major types of microtubule stabilizing proteins. Extensively studied MAPs are tau, MAP2 and MAP4 [4]. Tau and MAP2 are mainly expressed in neurons and take part in neuronal differentiation [4], [5]. MAP1B also known as MAP5 is involved in neuronal development [6], [7]. MAP3 is expressed in neurons as well as in various other tissues [8]. MAP4 is expressed in different types of cells [4], [5]. It was isolated from HeLa cells and has a role in mitosis [9]–[11]. MAP6 or Stable tubule only polypeptides (STOPs) are mainly expressed in adult neurons and regulate synaptic plasticity [12]. Another microtubule stabilizing protein that was isolated from HeLa cells is MAP7 which is also known as E-MAP-115 or ensconsin [9], [13], [14]. This protein is mainly expressed in polarized cells like epithelial cells, *Drosophila* oocytes and *Drosophila* and murine neurons [13], [15], [16]. Based on its cellular distribution during murine embryogenesis, it has been proposed to promote epithelial polarization and differentiation [16]. Interestingly overexpression of MAP7 has been reported to cause microtubule bundling [13]; however, its microtubule binding region did not alter microtubule dynamics on expression around four to ten times over the physiological level [17]. In recent studies, MAP7 has been shown to be essential for kinesin-1 driven functions [15], [18], [19].

Intriguingly, MAP7 is not homologous to the structural MAPs [13]. Also, while other structural MAPs lack in secondary structures in solution, MAP7 is predicted to have alpha helices near its N- and C-termini [13], [20], [21]. The alpha helices in both the regions are also annotated as potential coiled coil motifs and these are conserved across different species [15]. The N-terminal alpha helix is located within the positively charged microtubule binding region of the protein [13]. The C-terminal potential coiled coil motif is positioned in the MAP7 domain. MAP7 family has three other members namely MAP7 domain containing proteins 1, 2 and 3 or MAP7D1, MAP7D2 and MAP7D3 respectively. The members of MAP7 family are products of different genes [22]–[24]. Sequence alignment of these proteins with MAP7 using Protein BLAST suggests that each of the proteins shares greater than 50% sequence similarity with MAP7 near the N- and C-termini. Interestingly, the depletion of MAP7 but not other members of MAP7 family caused defects in nuclear positioning (a phenotype also caused on kineisn-1 depletion) in mouse myotubes [18]. This indicates that these proteins may not be redundant and that they potentially perform different functions in cells by interacting with different proteins.

**Figure 1:**
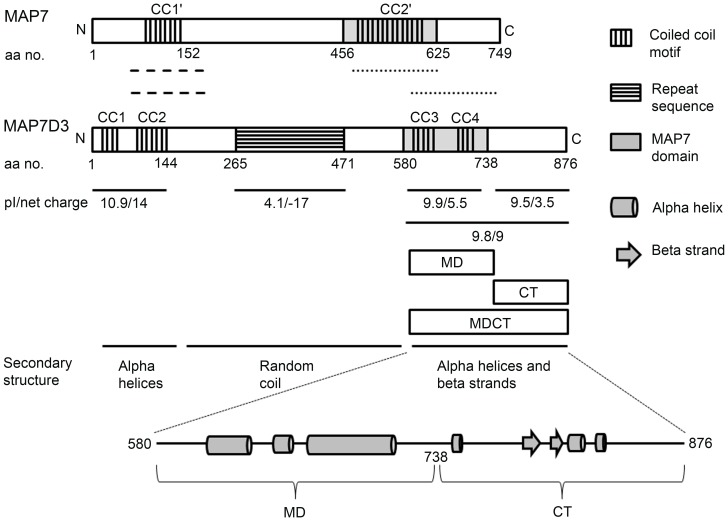
*In silico* characterization of MAP7D3. Protein BLAST of MAP7 and MAP7D3 identified two conserved regions located near the N- (− − −) and C- (……) termini of the proteins with 65 and 59% similarity, respectively. SMART identified coiled coil (CC) regions and MAP7 domain in both the proteins and a region of repeat sequences in MAP7D3 only. Net charges on different regions and their pIs were determined by EMBOSS Pepstats. Secondary structure prediction for MAP7D3 was done using SYMPRED.

MAP7D3 was identified during the proteome analysis of mitotic spindle of HeLa cells and shown to localize at the mitotic spindle upon overexpression [25]. It was shown to promote the assembly and stability of microtubules *in vitro* as well as in cells [25], [26]. MAP7D3 has two potential coiled coil motifs at the N-terminus and it was shown that these coiled coil motifs constitute the microtubule binding region of the protein, although the effects of the microtubule binding region on microtubule polymerization and stability are not known [26]. Interestingly, the N-terminal conserved region has a common function in the proteins MAP7 and MAP7D3. The role of MAP7 domain is known for MAP7 protein only, where it interacts with the C-terminal region of kinesin-1 [18]. The MAP7 domain is located between the residues 580 to 738 in MAP7D3, and it also contains two predicted coiled coil motifs (Figure 1).

The N-terminal conserved regions of all the MAP7 family members have similar predicted secondary structures and isoelectric points (pIs above 10.0), whereas the MAP7 domains have similar predicted secondary structures with variable pIs. For example, the pIs of MAP7 domains of MAP7, MAP7D1, MAP7D2 and MAP7D3 were calculated to be 7.6, 9.1, 5.8 and 9.9, respectively. Among these proteins, MAP7D3 has the most basic MAP7 domain. Interestingly, the MAP7 domain and the N-terminal microtubule binding region of MAP7D3 have two common features; 1) alpha helices with potential to form two coiled coil motifs and 2) basic pIs. The similarities between the N-terminal microtubule binding region and the MAP7 domain of MAP7D3 motivated us to hypothesize that the MAP7 domain of MAP7D3 has a microtubule binding activity. Secondary structure prediction of MAP7D3 suggested that the C-terminal tail of MAP7D3 has three short alpha helices and two beta strands (Figure 1). In agreement with the theoretically predicted secondary structural contents, both MAP7 domain and C-terminal tail were found to have secondary structures by far-UV CD analysis. The C-terminal tail of MAP7D3 is also positively charged with pI 9.5. Interestingly, the C-terminal tails of other MAP7 family members have pIs below 5.0. Keeping in mind the possibility that the MAP7 domain and the C-terminal tail together may form a unit with greater stability and higher positive charge than MAP7 domain or C-terminal tail separately, we checked the microtubule binding activity of three fragments namely MAP7 domain (MD), C-terminal tail (CT) and the longer fragment encompassing both MAP7 domain and C-terminal tail (MDCT). We report a novel microtubule binding region in MAP7D3. This region of MAP7D3 binds to microtubules through electrostatic interaction at the C-terminal tail of tubulin, promotes tubulin polymerization and prevents disassembly of preformed microtubules.

## Materials and Methods

### Materials

Taxol, bovine thrombin, GTP (guanosine triphosphate), mouse monoclonal anti-α-tubulin IgG, FITC (fluorescein isothiocyanate) conjugated anti-rabbit IgG, Alexa-568 conjugated anti-mouse IgG, alkaline phosphatase conjugated anti-mouse IgG and anti-rabbit IgG were procured from Sigma-Aldrich. Rabbit polyclonal anti-MAP7D3 IgG was obtained from Abcam. Isopropyl β-D-1-thiogalactopyranoside (IPTG) was obtained from Calbiochem and Ni-NTA agarose resin was obtained from Qiagen. All the other reagents used were of analytical grade and were from Sigma, Merck Millipore and Himedia (Mumbai, India) brands.

### Characterization of MAP7D3 using bioinformatics tools

Sequence similarity between MAP7 and MAP7D3 was determined using protein BLAST [27]. SMART (Simple Modular Architecture Tool), (smart. embl-heidelberg.de/) was used to identify the evolutionarily conserved domains in the proteins, MAP7 and MAP7D3 [28], [29]. The information obtained here was used to divide MAP7D3 into different fragments. These fragments were characterized with respect to their pIs and net charges using EMBOSS Pepstats (www.ebi.ac.uk/Tools/seqstats/emboss_pepstats/) [30]. SYMPRED consensus secondary structure prediction (www.ibi.vu.nl/programs/sympredwww/) was utilized to determine the secondary structure of MAP7D3.

### Cloning in bacterial expression vector

The fragments MD, CT and MDCT were cloned in bacterial expression vector pET-28a through standard cloning techniques. FLJ12649 (a kind gift from Dr. Erich A. Nigg) was used as the template for PCR amplification of the fragments [25]. Restriction sites for NheI and HindIII were introduced in the forward and the reverse primers respectively. NheI and HindIII treated PCR amplicons and vector were ligated and transformed into DH5-alpha *E. coli* competent cells. Clones were confirmed by DNA sequencing (Figure S1). The primers used for cloning are following:

Forward primer (MD and MDCT): 5′CTCTAGCTAGCATGATCTATGAAGAGTCTGG3′

Reverse primer (MD): 5′CCCCAAGCTTTTAGTCATGGCTGGATGTTTCTG3′

Forward primer (CT): 5′CTCTAGCTAGCATATATGAAGAGGCTGAGGC3′

Reverse primer (MDCT and CT): 5′CCCAAGCTTTTATTGTCTAAAGGTGTCTGAG3′

### Expression and isolation of recombinant proteins

The plasmids were transformed in BL21 (DE3) *E. coli* cells. The cells were grown in Luria-Bertani medium to reach OD_600_ to 0.6 and induction was done with 0.5 mM IPTG for 4 hours at 37°C. Since the desired constructs have 6xHis-tag at their N-termini, purification was achieved by affinity chromatography using Ni-NTA agarose resin according to the manufacturer's instructions. The protein fragments were dialyzed against 50 mM Sodium dihydrogen phosphate monohydrate (NaH_2_PO_4_) and 150 mM Sodium chloride (NaCl), concentrated to at least 40 µM and stored at −80°C. Quantification of protein was performed by method of Bradford [31]. Bovine serum albumin (BSA) was used as the standard. Recombinant rat tau 4R was expressed in BL21 (DE3) *E.coli* cells and purified as reported earlier [32]. Quantification of tau was done through densitometry of Coomassie blue R stained SDS-polyacrylamide gels using ImageJ software [33]. BSA was used as the standard.

### Thrombin treatment to remove 6xHis-tag

Every 1 mg of MDCT was incubated with 1U bovine thrombin at 4°C for 12 hours. The reaction was stopped by adding 2 mM phenylmethylsulfonyl fluoride (PMSF). The reaction mix was then incubated with Ni-NTA agarose resin to eliminate the 6xHis-tags generated by thrombin treatment. The unbound fraction was collected and concentrated to at least 40 µM and stored at -80°C. The removal of 6xHis-tag was checked by N-terminal sequencing of the thrombin treated MDCT.

### Tubulin isolation

Tubulin was isolated from goat brain by two cycles of polymerization in PEM buffer (50 mM PIPES, pH 6.8, 3 mM MgCl_2_, 1 mM EGTA) with 1 M L-glutamic acid and 10% (v/v) dimethyl sulfoxide (DMSO) at 37°C and depolymerization at 4°C in same buffer without glutamic acid and DMSO [34], [35]. Aliquots of tubulin were stored in PEM buffer with 0.1 mM GTP at -80°C. The goat brain tissues were purchased from a commercial butcher shop (Modern Mutton and Chicken Shop, Powai Market, Mumbai, India). Animal ethics committee approval is not required because selling goat brain tissues is legal in India [36].

### Circular dichroism (CD) analysis of MD, CT and MDCT

Secondary structures of MD, CT and MDCT were monitored in 10 mM phosphate buffer of pH 6.8 using JASCO J-810 spectropolarimeter. The far-UV CD spectra were analyzed using CDNN software [37].

### Sedimentation assay to study binding of MD, CT and MDCT to microtubules and their effects on tubulin assembly

To check the binding of MD, CT and MDCT to preformed microtubules, 20 µM tubulin was polymerized using 20 µM taxol in PEM buffer with 1 mM GTP for 15 min at 37°C. The polymers were diluted 5-fold in warm PEM buffer having 5 µM taxol in the presence of 4 µM MD, CT or MDCT and incubated at 25°C for 10 min. The samples were then subjected to centrifugation at 30000×g for 15 min at 30°C. The supernatant and pellet fractions were separated and analyzed by SDS-PAGE.

To find out effects of the different fragments on tubulin assembly, tubulin (10 µM) was incubated without and with 4 µM MD, CT, MDCT or MD and CT for 5 min in PEM buffer on ice and further incubated with 1 mM GTP for 10 min at 37°C. The samples were then centrifuged and the supernatant and pellet fractions were analyzed by SDS-PAGE.

To study the effect of MDCT on dilution induced disassembly of microtubules, 15 µM tubulin was polymerized with 8% DMSO in PEM buffer containing 1 mM GTP at 37°C for 15 min. Preformed polymers were then diluted 10-fold in warm PEM buffer without and with 1, 2 and 4 µM MDCT and incubated for an additional 10 min at 37°C. The polymers were collected by centrifugation and examined by SDS-PAGE. The protein bands were visualized by Coomassie staining and the band intensities were measured using ImageJ software.

### Kinetics of tubulin polymerization

Tubulin (10 µM) was incubated without and with 1, 2 and 4 µM MDCT in PEM buffer for 5 min on ice. Subsequently, 1 mM GTP was added to the sample and the assembly kinetics was monitored at 37°C by 90° light scattering (at 400 nm) using a fluorescence spectrophotometer (JASCO 6500). The effect of different concentrations (50, 75, 100 and 200 mM) of NaCl on MDCT-induced assembly of tubulin was also monitored by light scattering.

### Transmission electron microscopy

Tubulin (15 µM) was incubated without and with 5 µM MDCT in PEM buffer on ice for 5 min and then, polymerized for 10 min at 37°C in the presence of 1 mM GTP. Polymers were fixed using 0.5% glutaraldehyde, placed on Formvar carbon coated copper electron microscopy grids and stained with 2% uranyl acetate. To examine the morphology of the polymers, the fixed sample was diluted 5 times in warm PEM buffer and observed under a transmission electron microscope (FEI Tecnai-G^2^12, Philips, OR).

### Immunostaining to study the localization of purified MDCT on taxol stabilized microtubules

Tubulin (20 µM) was polymerized with 20 µM taxol for 10 min and then, incubated without and with 4 µM MDCT for an additional 10 min at 37°C. The protocol for *in vitro* immunostaining was modified from that described earlier [38]. Briefly, the samples were fixed with 0.1% glutardehyde for 5 min at 25°C and diluted 50 times in warm PEM buffer containing 5 µM taxol. The samples were layered on glass coverslips coated with 0.1% poly-L-lysine solution and then blocked with 4% BSA in phosphate buffered saline (PBS). Immunostaining was done by incubation with mouse anti-α-tubulin antibody and rabbit anti-MAP7D3 antibody for two hours followed by washing with PBS and incubation with Alexa-568 conjugated anti-mouse IgG and FITC conjugated anti-rabbit IgG secondary antibodies for an hour. The coverslips were mounted on glass slides and contemplated using a fluorescence microscope (Nikon Eclipse TE2000-U, Tokyo, Japan).

### Determination of dissociation constant of MDCT for microtubules

The dissociation constant for the binding interaction of MDCT and microtubules was determined by separating free MDCT from microtubule bound MDCT by centrifugation as described earlier [35]. Briefly, tubulin (20 µM) in the presence of 1 mM GTP was polymerized using 20 µM taxol for 15 min at 37°C and the preformed polymers were diluted 5 times in warm PEM buffer containing 2 µM taxol. Tubulin polymers (4 µM) were subsequently incubated with different concentrations (0.5, 1, 2, 3, 4, 6 and 8 µM) of MDCT for an additional 10 min at 25°C. Free MDCT was separated from the microtubule-bound MDCT by collecting the polymers through centrifugation. Pellets were dissolved in 1x SDS loading dye and analyzed by SDS-PAGE. Band intensity of MDCT corresponding to all the concentrations of MDCT was measured using ImageJ software. The background signal of MDCT was also estimated by pelleting MDCT exactly the same way in the absence of microtubules. The band intensity was measured as before and subtracted from the values of the respective bands obtained for MDCT on incubation with microtubules to get the corrected band intensity. Dissociation constant (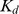)was calculated by GraphPad Prism 5 for Windows, GraphPad Software using the following equation:
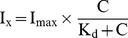



Where, I_x_ is the corrected band intensity of MDCT at a particular concentration *C* of MDCT, and I_max_ is the maximum value of corrected band intensity.

### Competition between tau and MDCT for binding to microtubules

Tubulin (20 µM) was polymerized in the presence of 1 mM GTP and 20 µM taxol in PEM buffer at 37°C for 15 min. Preformed microtubules were diluted 5 times in PEM buffer containing 5 µM taxol and 4 µM tau and incubated for an additional 10 min at 25°C. Then, the samples were incubated without or with 4 and 8 µM MDCT for another 10 min at 25°C. Microtubules were collected by centrifugation at 30000×g for 15 min at 30°C. Supernatant and pellet fractions were analyzed by SDS-PAGE. In the reciprocal experiment, microtubules were first incubated with 4 µM MDCT and then, incubated without or with 4 and 8 µM tau.

### Binding of MDCT to microtubules lacking the C-terminal tails of α- and β-tubulin subunits

Limited proteolysis of taxol stabilized microtubules using subtilisin cleaves the C-terminal tail of β-tubulin subunit alone or both α- and β-tubulin subunits [39], [40]. Tubulin (20 µM) polymerized in the presence of 20 µM taxol was treated with subtilisin, tubulin to subtilisin ratios being 1∶0.1 and 1∶1 (w/w) for 1 and 3.5 hours at 30°C respectively to remove the C-terminal tail of only β-tubulin (β_s_) and both α- and β-tubulin (α_s_β_s_). The reaction was terminated by adding 4 mM PMSF and microtubules were pelleted down. Microtubules without subtilisin treatment were also processed in the same way. The pellets were resuspended in PEM buffer containing 20 µM taxol. The three types of polymers were diluted 5 times in PEM buffer containing 5 µM taxol and incubated with 4 µM MDCT for 10 min at 25°C. Subsequently, microtubule-bound MDCT was separated from the unbound MDCT by centrifugation. The pellets were dissolved in 1x SDS loading dye and analyzed on 7.5% acrylamide/bis-acrylamide gels following a protocol described earlier [41]. The band intensity of MDCT in the pelleted polymers was obtained using ImageJ to calculate the relative amount of MDCT bound to subtilisin treated microtubules with respect to the untreated microtubules.

### Pull down assay

MDCT (100 µg) was incubated with 100 µl Ni-NTA agarose resin pre-equilibrated with binding buffer (50 mM NaH_2_PO_4_ and 50 mM NaCl) for 30 min on ice with continuous rocking. Unbound fraction was removed from the resin and the resin was washed with 1 ml wash buffer 1 (50 mM NaH_2_PO_4_, 50 mM NaCl and 10 mM imidazole) followed by the incubation of 250 µg precleared cell lysate (supernatant obtained after the incubation of cell lysate with 100 µl Ni-NTA resin) for an hour. Unbound fraction was removed and the resin was washed with 5 ml wash buffer 2 (50 mM NaH_2_PO_4_, 50 mM NaCl and 20 mM imidazole) and 5 ml wash buffer 3 (50 mM NaH_2_PO_4_, 50 mM NaCl and 30 mM imidazole) to remove non-specifically bound tubulin. As control, precleared cell lysate was incubated with free resin and treated in the same way as above. The resin was finally resuspended in 100 µl 1x SDS loading dye, boiled and centrifuged. 50 µl of the supernatant was run on 12% SDS polyacrylamide gel. Standard western blot protocol was followed to detect the proteins. MDCT was probed using rabbit anti-MAP7D3 antibody and tubulin was probed using mouse anti-α-tubulin antibody and the secondary antibodies were alkaline phosphatase conjugated. Nitro-blue tetrazolium (NBT) and 5-bromo-4-chloro-3-indolyl phosphate (BCIP) were used as substrates to develop the blot.

## Results

### Characterization of MD, CT and MDCT

**Figure 2:**
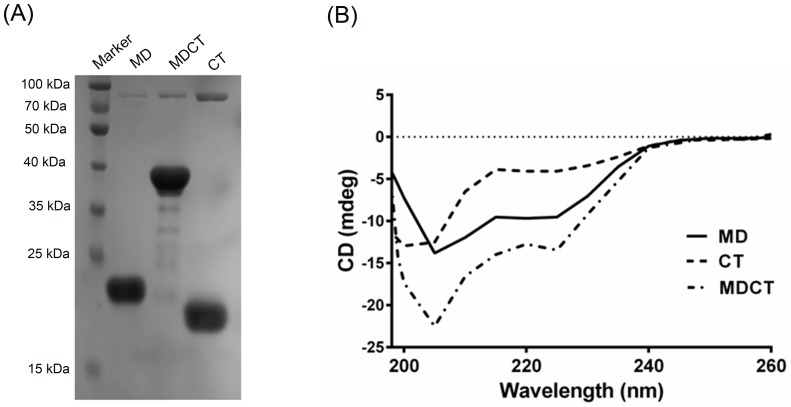
Characterization of MD, CT and MDCT. A, Purified MD, CT and MDCT were analyzed on 12% SDS-PAGE. The expected molecular weights of MD, CT and MDCT are 22, 18 and 37 kDa respectively. B, Far-UV CD spectra of MD, CT and MDCT are shown.

MD, CT and MDCT were isolated from BL21 (DE3) *E. coli* cells (Figure 2A). The theoretical molecular weights of MD, CT and MDCT are 22, 18 and 37 kDa respectively. Owing to the cloning strategy, these values also include the contribution from the 6xHis-tag and additional amino acid residues (including a thrombin cleavage site) located upstream to the actual sequence of the fragments. Secondary structures of the fragments were determined by far-UV circular dichroism spectroscopy (Figure 2B). An analysis of the spectral data indicated that in all the fragments the dominating secondary structures were alpha helix and random coil (Figure 2B). For example, MD had 38% alpha helices and 28% random coils, MDCT had 48% alpha helices and 22% random coils and CT had 31% alpha helices and 31% random coils, respectively. MD, CT and MDCT had 15, 18 and 11% beta strands and 16, 18 and 15% beta turns, respectively.

### MDCT displayed stronger ability to bind to microtubules than MD and CT

**Figure 3:**
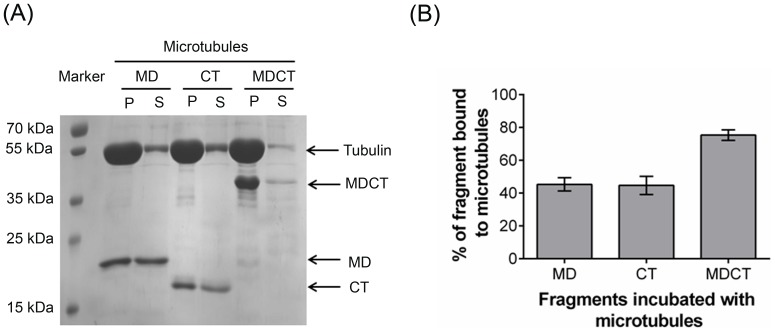
MDCT showed greater binding to microtubules as compared to MD and CT. A, preformed microtubules were incubated with MD, CT or MDCT for 5°C and microtubule bound protein fragments were separated by centrifugation. Polymeric (pellet, P) and soluble (supernatant, S) tubulin fractions were analyzed on 12% SDS-PAGE. B, Percent of each fragment bound to microtubules is plotted as mean ± SD of three independent sets.

To test if MD, CT and MDCT could bind to microtubules, preformed microtubules were incubated with MD, CT or MDCT and microtubule-bound MD, CT or MDCT were separated from the unbound fragments by sedimentation (Figure 3A). The amount of different fragments in the pellet and supernatant fractions were analyzed by densitometry (Figure 3B). It was found that 45±4, 45±6 and 75±3 percent of MD, CT and MDCT bound to the microtubules respectively, indicating that MDCT binds to microtubules with higher affinity as compared to MD and CT.

**Figure 4:**
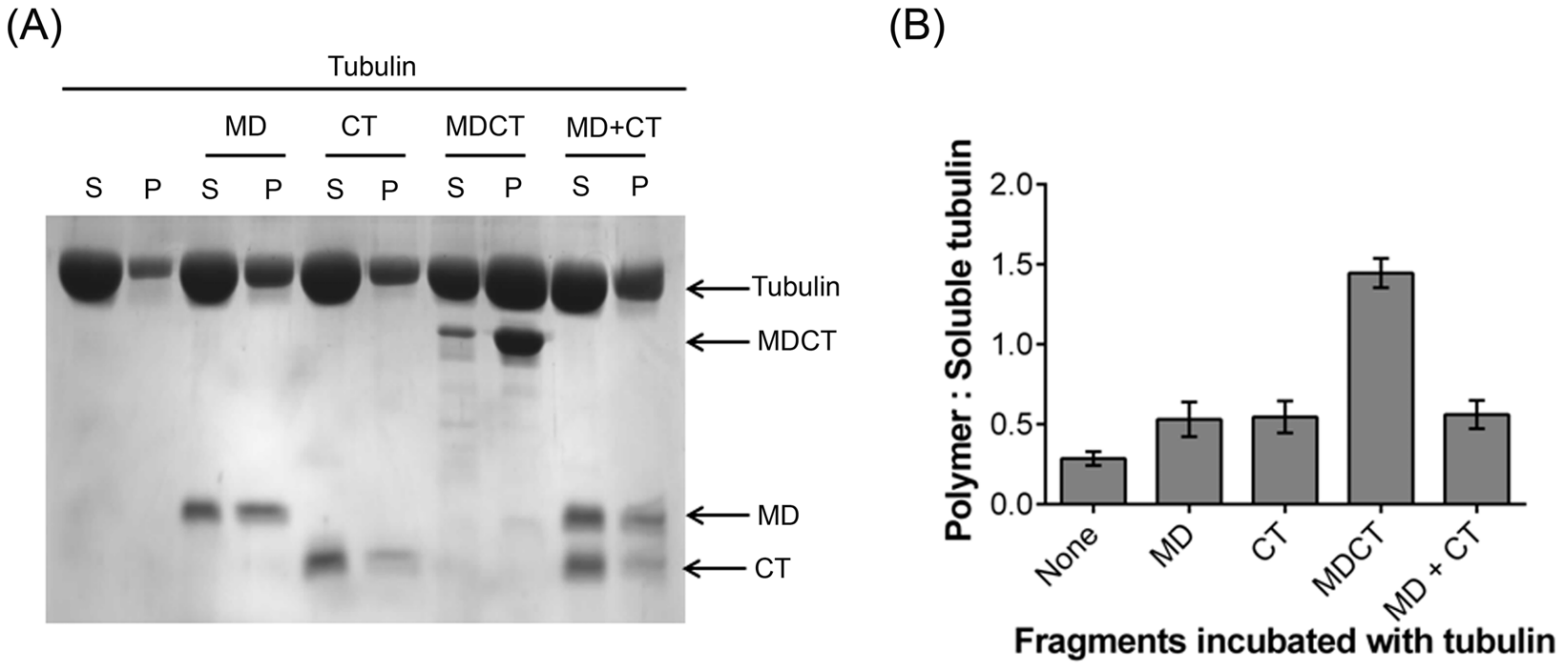
Effects of MD, CT and MDCT on the assembly of purified tubulin. A, Tubulin (10 µM) was polymerized in the absence and presence of 4 µM MD, CT, MDCT or MD and CT in PEM buffer containing 1 mM GTP. Supernatant and pellet fractions were analyzed using 12% SDS-PAGE. B, Ratio of polymerized tubulin to soluble tubulin in the presence of different fragments is plotted as mean ± SD of three sets of experiments.

Next, the effect of MD, CT, MDCT and MD with CT on the assembly of purified tubulin was estimated by determining the ratio of polymerized to soluble tubulin (Figure 4). The ratio in the absence of any of the fragments was found to be 0.3±0.04. In the presence of MD, CT and MDCT the ratio increased to 0.5±0.1, 0.5±0.1 and 1.4±0.1 respectively. When MD and CT were added together the ratio of polymerized to soluble tubulin was found to be 0.6±0.1, which was not significantly different from that when MD and CT were added separately suggesting that MD and CT show maximum promotion in tubulin assembly when present in a single unit as MDCT.

### MDCT promoted the assembly of purified tubulin

**Figure 5:**
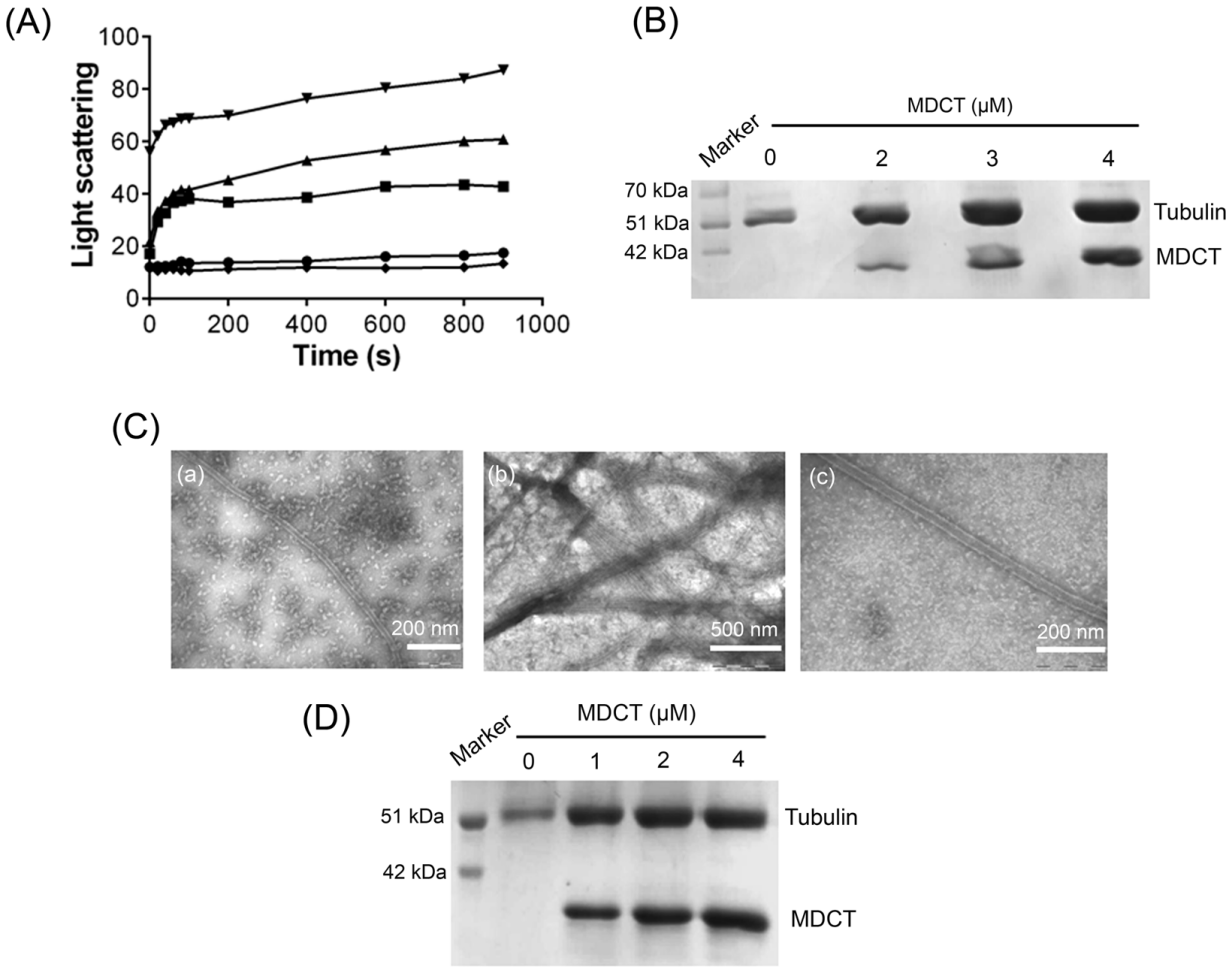
MDCT enhanced tubulin assembly in a concentration dependent manner *in vitro*. A, Effects of MDCT on the assembly kinetics of tubulin was monitored by light scattering at 400(10 µM) was polymerized in the absence (•) and presence of 1 (▪), 2 (▴) and 4 µM (▾) MDCT or 4 µM BSA (♦) in PEM buffer containing 1 mM GTP. B, The effect of MDCT on tubulin polymerization was determined by sedimentation assay. One of the three experiments is shown. C, MDCT caused an increase in the number of microtubules per field of view. Electron micrographs of polymerized tubulin in the absence (a) and in presence of MDCT (b) are shown. Polymers formed in presence of MDCT showed microtubule like morphology (c). D, MDCT stabilized microtubules against dilution induced disassembly. Preassembled microtubules were diluted in warm PEM buffer without or with 1, 2 and 4 µM MDCT and incubated for 10 min at 37°C. Microtubules were collected by centrifugation and the amount of microtubules recovered was estimated from a Coomassie-stained 12% SDS-PAGE. One of the three sets is shown.

After the initial observations that MDCT promoted tubulin assembly maximally, a set of experiments were performed to illustrate its effects on tubulin assembly. For these experiments, the N-terminal 6xHis-tag was cleaved from MDCT using thrombin and all the subsequent experiments were done using MDCT fragment without the His-tag. The effect of MDCT on the assembly kinetics of tubulin was monitored by light scattering (Figure 5A). MDCT increased the light scattering intensity of tubulin assembly in a concentration dependent manner indicating that it enhances tubulin assembly. BSA (4 µM) did not alter the light scattering signal indicating that the promotion in tubulin assembly in the presence of MDCT is not due to non-specific protein aggregation. Further, MDCT increased the amount of polymerized tubulin in a concentration dependent fashion (Figure 5B). The amount of polymerized tubulin as compared to the control (in the absence of MDCT) increased by 80±20, 110±20 and 120±10% in the presence of 1, 2 and 4 µM MDCT, respectively. In addition, electron microscopic analysis of the assembled polymers revealed that tubulin formed microtubules in the presence of MDCT (Figure 5C). Further, in the presence of MDCT, more microtubules were visible per field of view than in its absence.

### MDCT stabilized microtubules against dilution induced disassembly

Preformed microtubules were diluted 10-fold in PEM buffer without and with different concentrations of MDCT and incubated for 10 min at 37°C after which the polymers were collected by centrifugation. Analysis of the collected pellets demonstrated that higher amount of tubulin remained in the polymerized state in the presence of MDCT than without it (Figure 5D). For example, the amount of polymerized tubulin recovered in the presence of 1, 2 and 4 µM MDCT was 1.9±0.1, 2.3±0.1 and 2.4±0.2 fold of the polymerized tubulin recovered in its absence indicating that MDCT protected microtubules against dilution induced disassembly.

### MDCT localized along the length of microtubules

**Figure 6:**
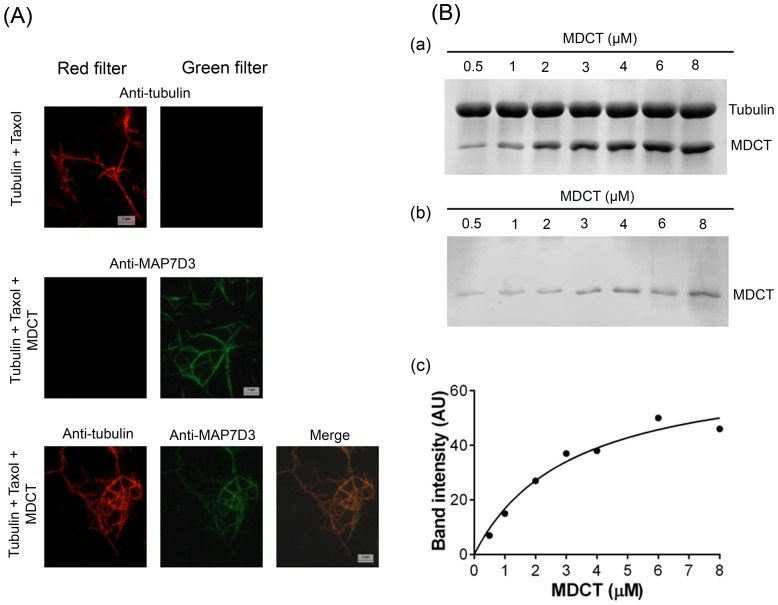
MDCT bound along the length of microtubules *in vitro*. A, tubulin (red) and MDCT (green) were detected using anti-α-tubulin and anti-MAP7D3 antibodies. Scale bar represents 2 µm. B, Determination of the dissociation constant for the interaction of MDCT and microtubules. Taxol stabilized microtubules were incubated with different concentrations (0.5 to 8 µM) of MDCT. Microtubule bound MDCT was estimated from the Coomassie blue stained 12% SDS-polyacrylamide gels. MDCT sedimented in the presence (a) and absence (b) of microtubules. A dissociation constant for the interaction of MDCT and microtubules was determined by fitting the data in a binding isotherm (c).

To study the localization of MDCT on microtubules, taxol stabilized microtubules were incubated with MDCT, fixed, diluted and co-immunostained with anti-α-tubulin and anti-MAP7D3 antibodies (Figure 6A). Immunostaining of taxol stabilized microtubules with anti-α-tubulin exhibited fluorescence only in the red channel and not in the green channel. Similarly taxol stabilized microtubules after incubation with MDCT and staining with anti-MAP7D3 antibodies showed fluorescence in the green and not in the red channel. This ruled out the likelihood of getting false positives due to bleed through. Immunofluorescence analysis of microtubules incubated with MDCT and co-immunostained with anti-α-tubulin and anti-MAP7D3 antibodies suggested that MDCT localized along the entire length of microtubules.

In addition, the binding of MDCT to reconstituted microtubules was examined using sedimentation assay. An apparent dissociation constant of MDCT for binding to microtubules was determined to be 3.0±0.5 µM (Figure 6B).

### MDCT bound to microtubules through electrostatic interaction

**Figure 7:**
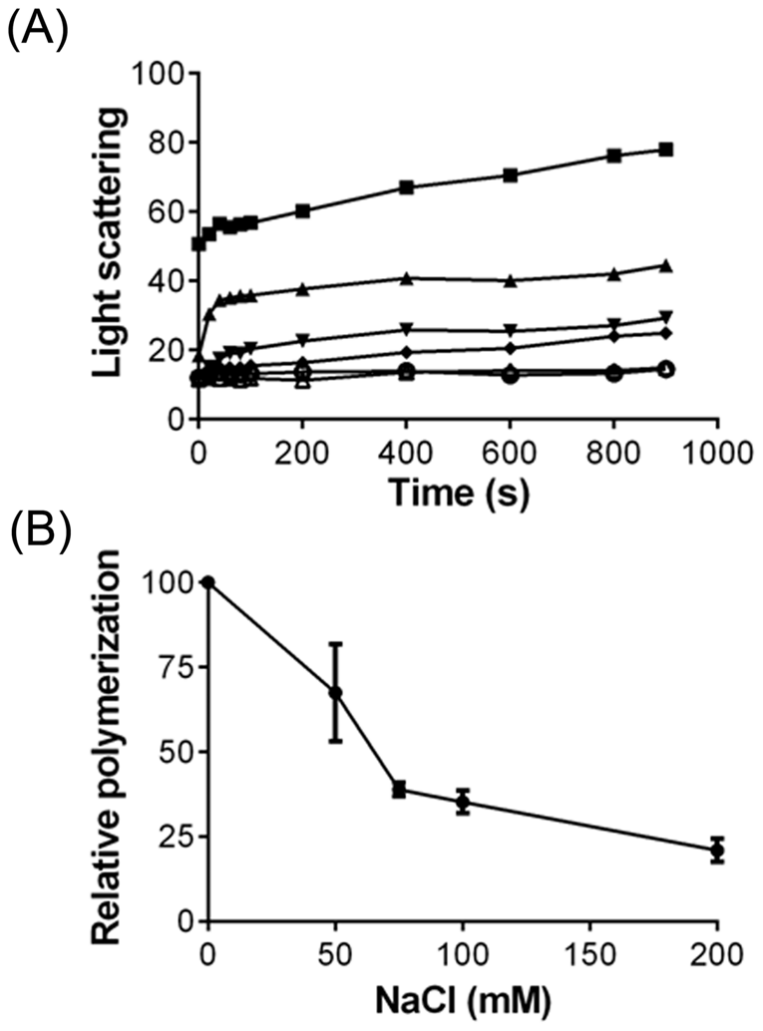
Effects of ionic salt on the MDCT-induced assembly of tubulin. A, Tubulin (10 µM) was incubated with 4 µM MDCT in PEM buffer in the absence (▪) and presence of 50 (▴), 75 (▾), 100 (♦) and 200 (o) mM NaCl for 5 min on ice. Subsequently, 1 mM GTP was added to the reaction mixtures and the assembly kinetics was monitored at 37°C. The assembly of tubulin (10 µM) without MDCT and NaCl (Δ) was also monitored. B, NaCl inhibited MDCT-induced assembly of tubulin. The data represent average ± SD of three independent sets.

NaCl produced inhibitory effect on the MDCT-induced assembly of tubulin (Figure 7). For example, in the presence of 50, 75, 100 and 200 mM NaCl, the light scattering signal after 900 seconds was found to be 68±14, 39±2, 35±3.3, 21±3.4 percent of that without additional NaCl (Figure 7B). In fact, at 200 mM NaCl concentration the light scattering signal reduced to the same value as for tubulin without MDCT indicating that the interaction between microtubules and MDCT is electrostatic in nature (Figure 7A).

### MDCT shared a binding site with tau on microtubules

**Figure 8:**
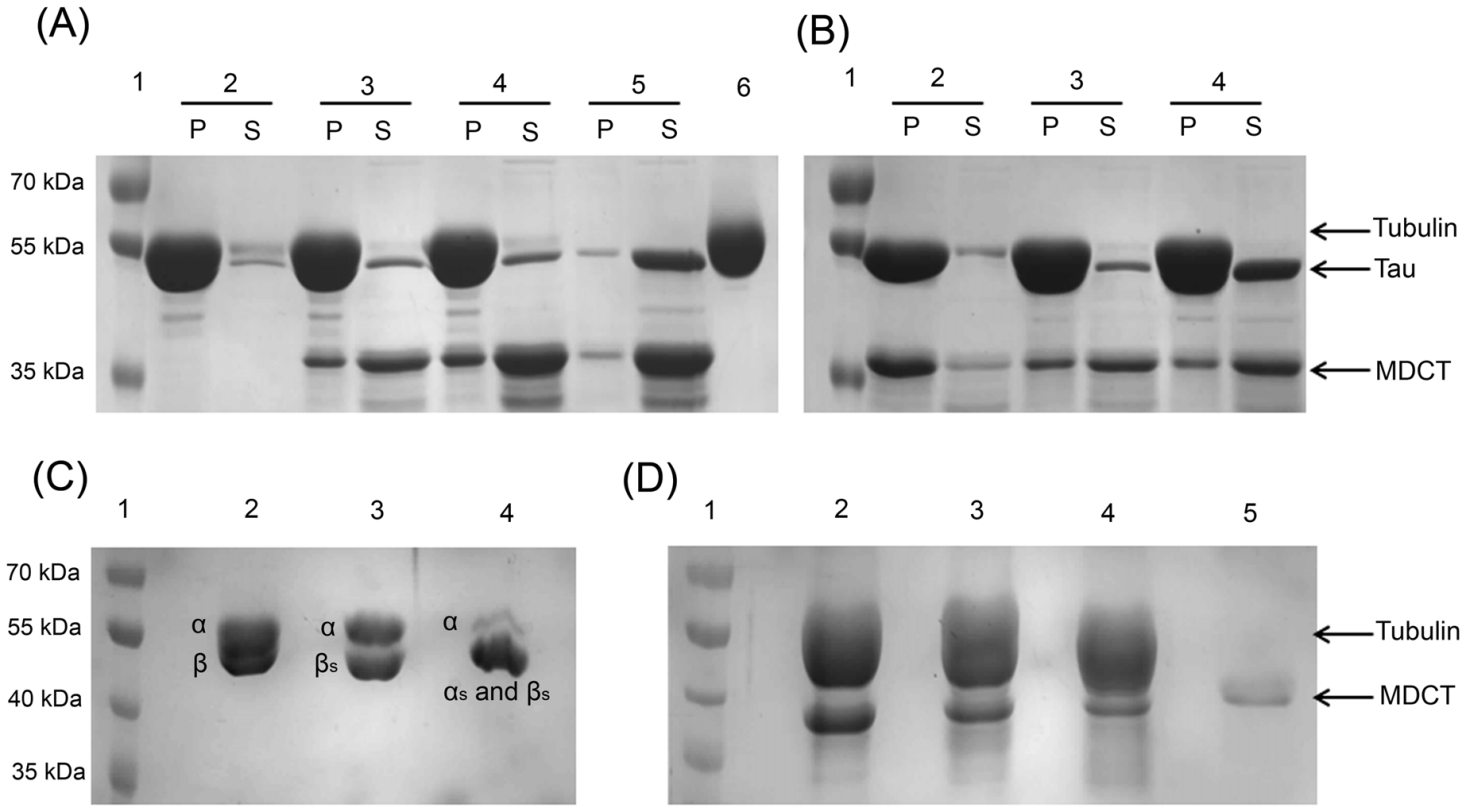
MDCT bound at the C-terminal tail of tubulin subunits in microtubules. A, MDCT shares binding site with tau on microtubules. Taxol stabilized microtubules were incubated with 4 µM tau for 10 min at 25°C and then, incubated without (2) or with 4 (3) or 8 µM (4) MDCT for an additional 10 min. Polymers were collected through centrifugation. A mixture of tau and MDCT without microtubules (5) was also treated the same way. Supernatant (S) and pellet (P) fractions were analyzed by SDS-PAGE. Only tubulin (6) was also run in the same gel to compare its position with tau. B, Taxol stabilized microtubules were first incubated with 4 µM MDCT for 10 min at 25°C and then, incubated without (2) or with 4 (3) and 8 µM (4) tau for another 10 min. The samples were analyzed as described previously. C, Taxol stabilized microtubules were treated with subtilisin to cleave C-terminal tail of β-tubulin alone (αβ_s_) (lane 3) and C-terminal tail of both α and β tubulin (α_s_β_s_) (lane 4) and analyzed on 7.5% SDS-polyacrylamide gel. Lanes 1 and 2 have protein marker and taxol stabilized microtubules without subtilisin treatment, respectively. D, MDCT showed lesser binding to subtilisin treated microtubules. Microtubules composed of αβ, αβ_s_ or α_s_β_s_ tubulin dimers were incubated with MDCT and centrifuged to pellet the microtubules. Pellets were analyzed on 7.5% polyacrylamide gel to check the amount of MDCT bound to αβ, αβ_s_ or α_s_β_s_ containing microtubules, lanes 2, 3 and 4, respectively. Lanes 1 and 5 respectively, have protein molecular weight marker and MDCT that pelleted down in the absence of microtubules. One of the three sets is shown.

The structural MAPs have overlapping binding sites on microtubules, mainly located in the negatively charged C-terminal tails of α- and β-tubulin subunits, which protrude from the surface of the filament [42], [43]. To examine whether MDCT could bind at the same site on tubulin, competition experiments with tau for binding to microtubules were performed. Preformed microtubules were first incubated with 4 µM tau and then, with 4 or 8 µM MDCT (Figure 8A). Subsequently, microtubule pellets were collected and the pellet and supernatant fractions were analyzed to estimate the microtubule-bound tau and MDCT. Tau was found to co-sediment with microtubules in the absence of MDCT. Also, when MDCT was added to microtubules that had already been incubated with tau most of the tau could pellet with microtubules, whereas most of the MDCT remained in the supernatant. Even at 8 µM concentration of MDCT, only a small fraction of MDCT was found to bind to the microtubules in the presence of tau. The results suggested that either MDCT shared its binding site with tau on the microtubules or tau brought a conformational change in the microtubules so that MDCT could not access its binding site. To validate the two possibilities the reciprocal experiment was performed, where microtubules were first incubated with MDCT and tau was added later (Figure 8B). In the absence of tau most of MDCT could pellet with microtubules. However, when tau was added to the microtubules that were already bound with MDCT, only a small fraction of MDCT was obtained in the pellet fraction, while most of the tau was pelleted with the microtubules. The result suggested that tau replaced the microtubule-bound MDCT thus, providing evidence in support of the first possibility that tau and MDCT share the binding site on the microtubules. This also showed that affinity of MDCT for microtubules is lower than that of tau which is consistent with the dissociation constant of MDCT for microtubules determined in this study.

To examine whether MDCT binds at the C-terminal tail of tubulin, taxol stabilized microtubules were treated with subtilisin to prepare microtubules composed of αβ_s_ and α_s_β_s_ subunits (Figure 8C). Microtubules composed of αβ subunits were used as control. The three types of microtubules were incubated with MDCT and the polymer-bound MDCT were separated through sedimentation. The analysis of the pellets by SDS-PAGE suggested that the amount of MDCT bound to microtubules was less in the case of αβ_s_-microtubules and α_s_β_s_-microtubules in comparison to αβ-microtubules (Figure 8D). For example, MDCT bound to αβ_s_- and α_s_β_s_-microtubules was reduced to 44±12 and 12±11%, respectively, as compared to its binding to αβ-microtubules. The findings indicated that MDCT binds to the C-terminal tail of tubulin and probably to the C-terminal tails of both α- and β-tubulin subunits.

### MDCT could pull down tubulin from HeLa cell extract

**Figure 9:**
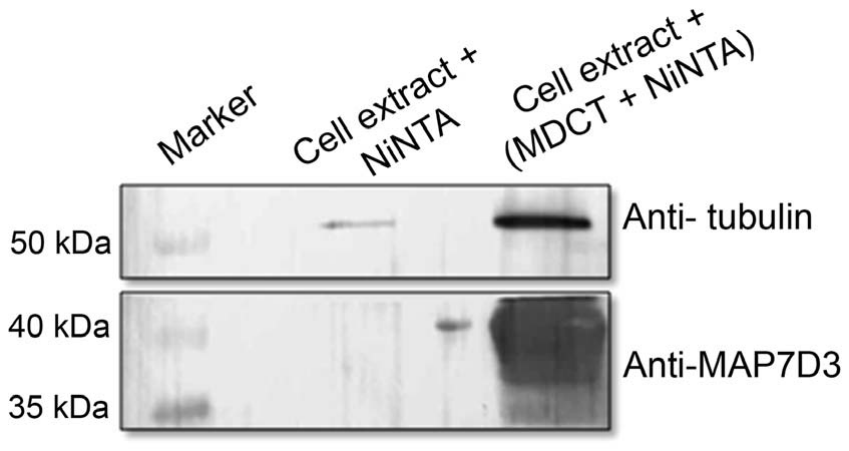
MDCT bound to tubulin in HeLa cell extract. Tubulin was precipitated from the HeLa cell extract using MDCT bound with Ni-NTA agarose resin as bait. Anti α-tubulin and anti- MAP7D3 antibodies were used to detect tubulin and MDCT, respectively.

Whole cell lysate of HeLa cells was incubated with free Ni-NTA resin or 6xHis-MDCT bound Ni-NTA resin and the resin bound fraction was probed for tubulin through western blot. An intense band of tubulin was obtained in case of His-MDCT bound resin (Figure 9). However, with the free resin a faint band of tubulin appeared in the blot. This suggested that MDCT can bind to tubulin in the HeLa cell extract.

## Discussion

In this study, we have identified a novel microtubule binding region, MDCT in the MAP7D3 protein. This region is constituted by the MAP7 domain (MD) and the C-terminal tail (CT) of the protein. MDCT bound to reconstituted microtubules, promoted microtubule polymerization and stability *in vitro*. It could pull down tubulin from the HeLa cell extract. Further, MDCT was found to share its binding site on microtubules with tau.

### Intramolecular interaction between MD and CT regions appear to be essential for the microtubule binding and polymerization promoting activity of MDCT

Interestingly, as compared to MDCT its two components, MD and CT showed weaker binding to microtubules. Assuming a linear relationship, the binding free energy of MDCT would be equal to the sum of the binding energies of MD and CT, which should increase the binding affinity of MDCT for microtubules as compared to its individual components. These fragments also enhanced the polymerization of tubulin mildly as compared to MDCT. MD and CT are positively charged fragments and the total charge adds up when they are present together as MDCT, which might be the reason for the stronger binding of MDCT to microtubules as compared to MD and CT individually. This is supported by the finding that MDCT-induced tubulin polymerization was strongly inhibited by NaCl, indicating that similar to the structural MAPs, the interaction between MDCT and tubulin is predominantly electrostatic [44], [45]. The charge based interaction is essential for the MDCT-induced tubulin polymerization. However, it may not be sufficient for the same, as MD and CT separately as well as in combination as individual fragments were significantly less effective in increasing microtubule polymerization than MDCT, suggesting that MD and CT must be present as a single fragment to form a functional unit with maximal activity.

MDCT might form a tertiary structure through intramolecular interactions between MD and CT regions that can bind to microtubules and promote their polymerization more strongly. Intramolecular interactions have been shown to have role in the regulation of microtubule binding and polymerizing activity of tau [46]. The proline rich region of tau alone has no significant effect on microtubule assembly; however, when conjugated to the repeat region it enhances the microtubule polymerizing ability of the repeat region by 10-fold. The structural MAPs have open structure in solution [20], [21]. However, there are indicative evidences in favor of a folded structure of the repeat regions in the microtubule bound state [47], [48]. Also the tubulin/microtubule binding regions of the families of plus end tracking proteins like EB1, CLIP 170, and XMAP215 are formed by different conserved domains, which are present in multiple copies [3], [49]. Each domain has a unique structure that creates the interface for interaction with tubulin [49], [50]. Similarly, MD and CT regions, owing to the requirement of a proper architecture for binding to microtubules, might have a greater affinity for the microtubules and a higher microtubule polymerization promoting activity when present in a single polypeptide as in MDCT. Further, the dissociation rate of MDCT may also be lower than MD and CT because the dissociation of MDCT needs to break more interactions than MD and CT, which would contribute in increasing the binding strength.

### MDCT decorates microtubules by binding at the C-terminal tails of α- and β-tubulin subunits

MAP7D3 was shown to localize along the length of the interphase microtubules and throughout the mitotic spindle on over expression in 293 T cells and HeLa cells respectively [25], [26]. On examining the localization of MDCT on the taxol stabilized microtubules *in vitro*, we found that it also localized along the entire length of the microtubules. Structural MAPs bind to microtubules by interacting with the C-terminal tails of α- and β-tubulin subunits [42], [43]. The binding sites of MAP7D3 and other members of MAP7 family on the microtubules have not been studied. The results of the competition experiment indicated that MDCT fails to bind microtubules that were incubated with 4R tau. Tau also displaced bound MDCT from the microtubules indicating that MDCT shares its binding site with tau on the microtubules. The apparent dissociation constant of MDCT for binding to microtubules was found to be 3.0±0.5 µM, which is several times higher than the dissociation constant of 4R tau (0.13±0.02 µM) suggesting that MDCT binds to microtubules with an affinity much lower than that of tau [46]. The microtubule binding regions of bovine brain MAP2 and MAP4 show apparent dissociation constant 1.1±0.1 and 0.27 µM respectively [51], [52]. Furthermore, the binding of MDCT to microtubules lacking C-terminal tail of β-tubulin subunit and α- and β-tubulin subunits was decreased by 56 and 88% as compared to control microtubules further suggesting that MDCT binds at the C-terminal tail of tubulin. These results also suggested that similar to the structural MAPs, MDCT binds to both α- and β-tubulin subunits. The binding of MDCT at the negatively charged C-termini of the α- and β-tubulin subunits might decrease or overcome the repulsive forces between the tubulin dimers thereby promoting microtubule polymerization.

### MDCT may have a role in determining the effects of MAP7D3 on microtubules

MDCT and the previously reported microtubule binding region of MAP7D3 are located at the C- and N-termini of the protein, respectively. The two microtubule binding regions are separated by a long stretch of 436 amino acid residues. In the case of structural MAPs and plus end tracking proteins multiple tubulin/microtubule binding regions are closely spaced which contribute differentially to the overall activity of the protein [49], [53]–[57]. In the PAR1 binding protein MTCL1, two microtubule binding regions have been reported near the N and C-termini [58]. Each region can bind to microtubules in the absence of the other; however, only the N-terminal microtubule binding region in conjugation with the two following coiled coil motifs is enough to cause the bundling of apicobasal microtubules in polarized epithelial cells. Role of the C-terminal microtubule binding region is not known. In the case of MAP7D3 as well, it is difficult to define the significance of two distantly spaced microtubule binding regions based on the data available from work done by us and work reported by Sun et al (2011) [26]. It is possible that both the regions contribute to the microtubule binding property of MAP7D3 and cause the microtubule bundling by crosslinking the microtubules as observed on overexpression of MAP7D3 in HeLa cells [25]. Also, the presence of two microtubule binding regions might enhance the affinity of the protein for the microtubules. Another interesting possibility is that the binding of each region to the microtubules is regulated independently to fine tune the affinity of MAP7D3 for microtubules during different stages of cell cycle according to the requirement of the cell.

## Conclusion

MAP7D3 is a newly identified protein and information necessary to understand the function and significance of this protein is not yet available. The present work has identified a new microtubule binding region in MAP7D3, formed by the conserved MAP7 domain and the C-terminal tail, which can bind to microtubules and promote their assembly and stability. The results of this study together with the earlier finding that MAP7D3 is present in the mitotic spindle proteome indicate that it may have an important role in the formation of the mitotic spindle.

## Supporting Information

Figure S1 **Nucleotide sequences of MD, CT and MDCT.** Clones containing the fragments MD, CT and MDCT were subjected to DNA sequencing. The underlined nucleotides in the starting and end of the sequences are the restriction sites for NheI and HindIII, the restriction enzymes that were used for cloning the fragments into pET-28 a vector. The nucleotide sequences are from 5′ to 3′ end.
(TIF)
