# Supplementary figures and images for: C-Terminal Region of MAP7 Domain Containing Protein 3 (MAP7D3) Promotes Microtubule Polymerization by Binding at the C-Terminal Tail of Tubulin
Source: PLoS One. 2014 Jun 13;9(6):e99539. doi: 10.1371/journal.pone.0099539 (PMC4057234; doi:10.1371/journal.pone.0099539)

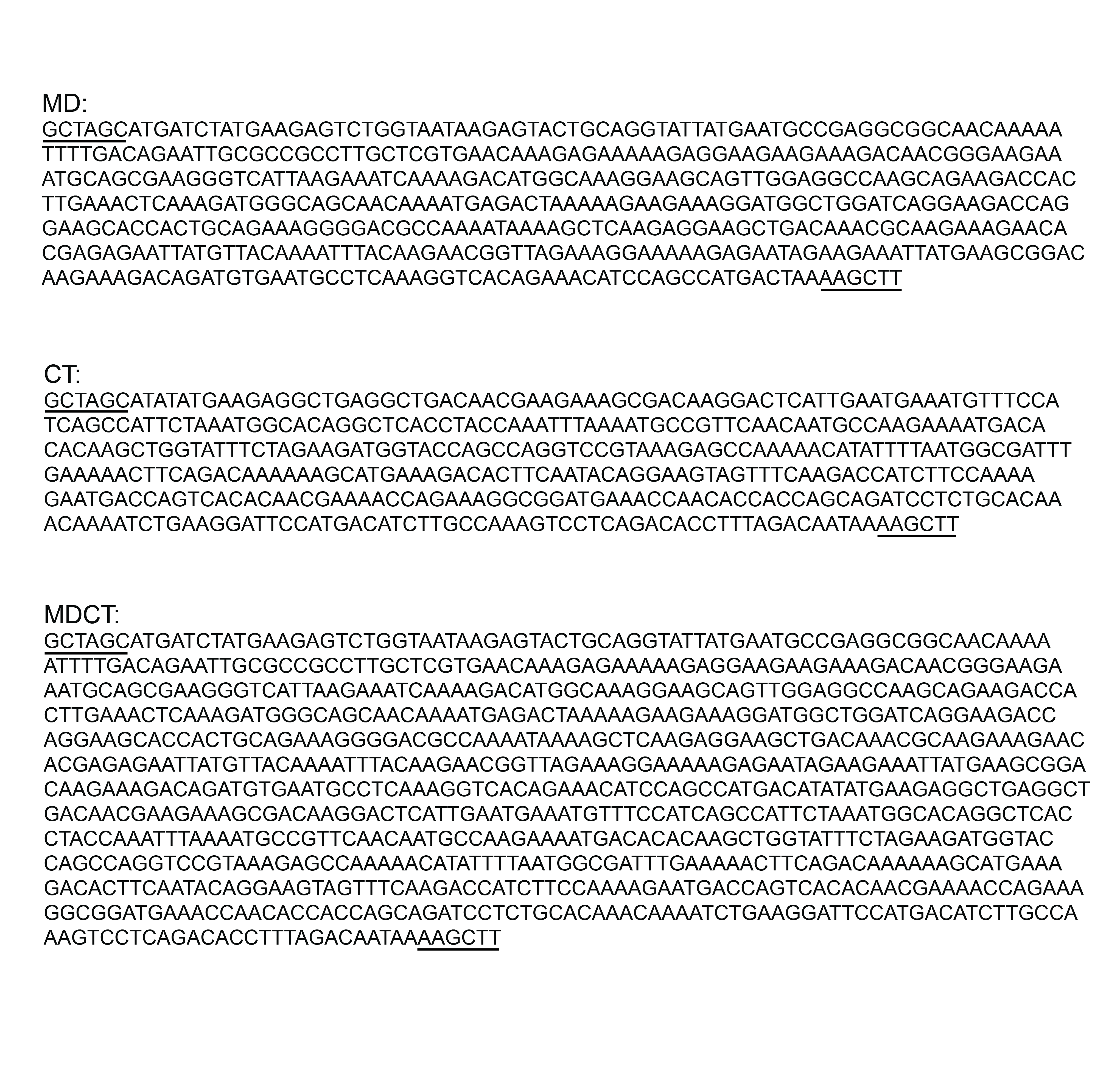

Supplement: Figure S1 — Nucleotide sequences of MD, CT and MDCT. Clones containing the fragments MD, CT and MDCT were subjected to DNA sequencing. The underlined nucleotides in the starting and end of the sequences are the restriction sites for NheI and HindIII, the restriction enzymes that were used for cloning the fragments into pET-28 a vector. The nucleotide sequences are from 5′ to 3′ end. (TIF) [file pone.0099539.s001.tif]
